# A Case of Unresectable Ampulla of Vater Carcinoma Successfully Treated with Conversion Surgery Following Sequential Systemic Chemotherapy Including Durvalumab

**DOI:** 10.70352/scrj.cr.26-0018

**Published:** 2026-06-19

**Authors:** Tatsuhiro Araki, Ryo Muranushi, Kohji Takagi, Toru Watanabe, Noriko Okuno, Mina Fukasawa, Nana Kimura, Yuuko Tohmatsu, Yoshihiro Shirai, Kazuto Shibuya, Isaku Yoshioka, Kenichi Hirabayashi, Ichiro Yasuda, Tsutomu Fujii

**Affiliations:** 1 Department of Surgery and Science, Faculty of Medicine, Academic Assembly, University of Toyama, Toyama, Toyama, Japan; 2 Department of Diagnostic Pathology, Faculty of Medicine, Academic Assembly, University of Toyama, Toyama, Toyama, Japan; 3 Third Department of Internal Medicine, Faculty of Medicine, Academic Assembly, University of Toyama, Toyama, Toyama, Japan

**Keywords:** unresectable ampulla of Vater carcinoma, durvalumab and gemcitabine plus cisplatin, conversion surgery

## Abstract

**INTRODUCTION:**

The criteria for indications and postoperative treatment strategies for conversion surgery in patients with unresectable ampulla of Vater carcinoma with distant metastasis have not been clarified. We present an unusual case of a patient with initially unresectable ampulla of Vater carcinoma with multiple liver metastases who underwent conversion surgery after treatment with durvalumab and gemcitabine plus cisplatin.

**CASE PRESENTATION:**

A 69-year-old male was found to have ampulla of Vater carcinoma with multiple liver metastases. A diagnosis of unresectable ampulla of Vater carcinoma was made, and the treatment plan was chemotherapy. After 12 cycles of gemcitabine, cisplatin, and S-1, the tumor did not shrink. Six cycles of gemcitabine, cisplatin, and durvalumab were then administered. The metastases were obscured on imaging; however, micrometastases were observed on the liver surface by staging laparoscopy. Six additional cycles of gemcitabine, cisplatin, and durvalumab were administered, and the disappearance of metastases was confirmed by staging laparoscopy. The patient then underwent conversion surgery with subtotal gastric-preserving pancreaticoduodenectomy. Pathology revealed a complete response, and radical resection was successfully performed. Immunostaining of tissue collected during endoscopic ultrasound-guided acquisition revealed less than 1% expression of programmed death ligand-1.

**CONCLUSIONS:**

Sequential systemic chemotherapy including durvalumab is suggested as a useful future treatment option and may contribute to conversion surgery for unresectable ampulla of Vater carcinoma.

## Abbreviations

CA19-9
: carbohydrate antigen 19-9
CEA
: carcinoembryonic antigen
GC
: gemcitabine plus cisplatin
GCD
: durvalumab in combination with gemcitabine and cisplatin
GCS
: S-1 in combination with gemcitabine and cisplatin
GS
: gemcitabine plus S-1
PD-L1
: programmed death ligand-1
UICC
: Union for International Cancer Control

## INTRODUCTION

The most common initial symptom of ampulla of Vater carcinoma is obstructive jaundice, which is detected at a relatively early stage among biliary tract cancers. Cancers at this stage are resectable in half of the cases.^1)^ In cases of ampulla of Vater carcinoma, the median overall survival after resection is 70 months, which is a more favorable prognosis than that of distal bile duct cancer, where the median overall survival is 41 months.^2)^ On the other hand, the 5-year survival rate for stage IV ampulla of Vater carcinoma is extremely poor (11.5%).^3)^ Previous first-line treatment options for unresectable ampulla of Vater carcinoma include GC (ABC-02; phase III)^4)^ and GS (JCOG1113; phase III).^5)^ In 2022, an interim analysis of the phase III TOPAZ-1 trial revealed the efficacy of GCD for treating patients with metastatic, unresectable, or recurrent biliary tract cancer.^6)^ In December 2022, this regimen was approved in Japan for treating unresectable biliary tract cancer.^6)^ In 2023, the phase III MITSUBA trial also reported on the efficacy of GCS for unresectable biliary tract cancer.^7)^ The advent of GCD and GCS therapies has expanded the range of treatment options to achieve conversion surgery for unresectable biliary tract cancer.

However, an optimal treatment plan has yet to be established for ampulla of Vater carcinoma with distant metastases, particularly regarding conversion surgery. This case study presents a patient with unresectable ampulla of Vater carcinoma with multiple liver metastases who underwent conversion surgery after receiving GCD therapy in addition to GCS therapy.

## CASE PRESENTATION

We present the case of a 69-year-old male whose chief complaint was jaundice. The patient developed chronic glomerulonephritis at age 40, and his creatinine clearance was 51 mL/min. Blood tests to assess tumor marker levels on the first day of the study revealed a CEA concentration of 6.3 U/mL and a CA19-9 concentration of 103 U/mL, both of which were high. Dynamic CT revealed a 15 × 13-mm mass lesion in the ampulla of Vater with bile duct dilatation extending into the intrahepatic bile duct (**Fig. 1A** and **1B**). There was no obvious lymph node enlargement. Endoscopic findings revealed a tumor primarily located at the ampulla of Vater, which was accompanied by a significant enlargement of the papillary structure. Endoscopic US-guided tissue acquisition of the tumor indicated adenocarcinoma (**Fig. 1C**). Because the tumor was confined to the papilla and there were no obvious lesions in the surrounding duodenal mucosa, a clinical diagnosis of ampulla of Vater carcinoma was made. Gadolinium ethoxybenzyl diethylenetriamine pentaacetic acid-enhanced MRI revealed multiple low-signal nodules in the hepatocellular phase, which were evaluated as multiple liver metastases (**Fig. 1D**). The patient had unresectable ampulla of Vater carcinoma with multiple liver metastases and a preoperative diagnosis of cT3b, cN0, cM1(H1), and cStageIV (UICC 8th Edition).

**Fig. 1:**
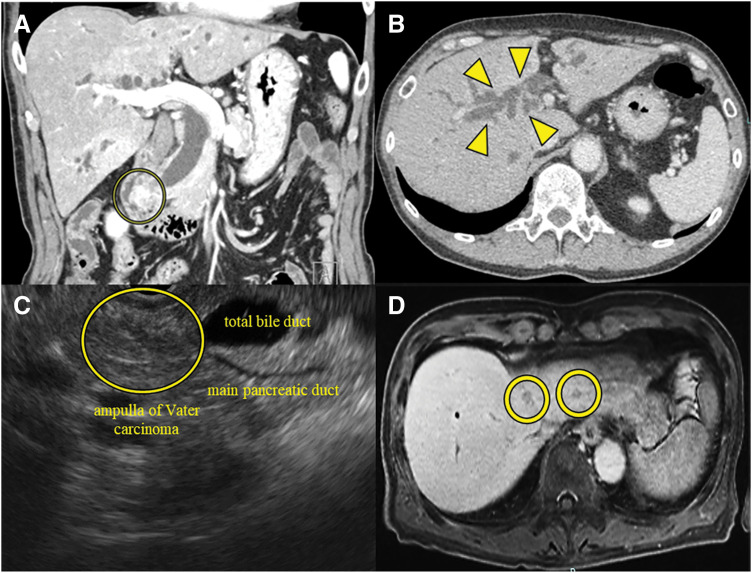
(**A**) Contrast-enhanced CT image of ampulla of Vater carcinoma. Yellow circle, tumor. (**B**) The bile ducts are dilated. Dilation extends into the intrahepatic bile ducts. (**C**) Endoscopic ultrasonography revealed that the common bile duct and the main pancreatic duct were dilated. (**D**) The hepatocellular phase of EOB-MRI revealed multiple low-signal nodules. EOB-MRI, gadolinium ethoxybenzyl diethylenetriamine pentaacetic acid-enhanced magnetic resonance imaging

With the placement of a bile duct stent, rapid drainage was achieved. Serum bilirubin levels returned to normal. GCS was introduced as the initial treatment. Since creatinine clearance was 51 mL/min, the cisplatin dose was reduced by 25% (31 mg, 25 mg/m^2^), and S-1 (100 mg) and gemcitabine (1600 mg) administration was started (**Fig. 2**). There were no apparent adverse events. No worsening of adverse events, including renal impairment, was observed during the period of cisplatin use; therefore, discontinuation or interruption of treatment was not necessary. After 12 cycles of treatment with gemcitabine, cisplatin, and S-1, the tumor failed to shrink. Following GCS therapy, the primary tumor had nearly disappeared due to shrinkage. Based on this, we estimate that it has shrunk by more than 90%. On the other hand, although GCS therapy resulted in a reduction in the size of the liver metastases, the rate of reduction was less than 30% (**Figs. 3A**, **3B** and **4A**, **4B**). The final assessment of treatment response was a diagnosis of stable disease (**Figs. 3A**, **3B**, and **4A**, **4B**). As imaging studies using GCS revealed residual liver metastases that were classified as stable disease, the regimen was switched to GCD in the hope of achieving further antitumor effects and enabling conversion surgery (**Figs. 3C**, **3D** and **4C**, **4D**). After 6 cycles of treatment with gemcitabine (1600 mg), cisplatin (31 mg; 25 mg/m^2^), and durvalumab (1500 mg), the CEA level was 5.6 U/mL and the CA19-9 level was 35 U/mL. (**Fig. 2**). Gadolinium ethoxybenzyl diethylenetriamine pentaacetic acid-enhanced MRI obscured the liver metastases (**Fig. 5A**). Staging laparoscopy was performed at this time, and multiple white nodules were found on the segment 2, 3 and 4 surfaces of the liver; after partial resection, adenocarcinoma was detected (**Fig. 5B**). After 6 more cycles of GCD therapy, the tumor marker levels normalized. Imaging studies revealed that the liver metastases were obscured. Second-stage laparoscopy was performed, and all remaining white nodules were partially resected, none of which showed malignant findings. The patient experienced no apparent adverse events due to GCD therapy. The patient was downstaged with disappearance of liver metastases, and tumor markers were confirmed to be negative. The preoperative diagnosis was ycT1b, ycN0, ycM0, ycStageIB (UICC 8th Edition), and conversion surgery was considered feasible. The surgical method was subtotal gastric-preserving pancreaticoduodenectomy-II. We adopted the T-anastomosis technique, developed at our institution, for choledochojejunostomy. This technique has been shown to significantly reduce the incidence of midterm postoperative cholangitis.^8)^ The time period between the initial diagnosis and conversion surgery was 17 months.

**Fig. 2:**
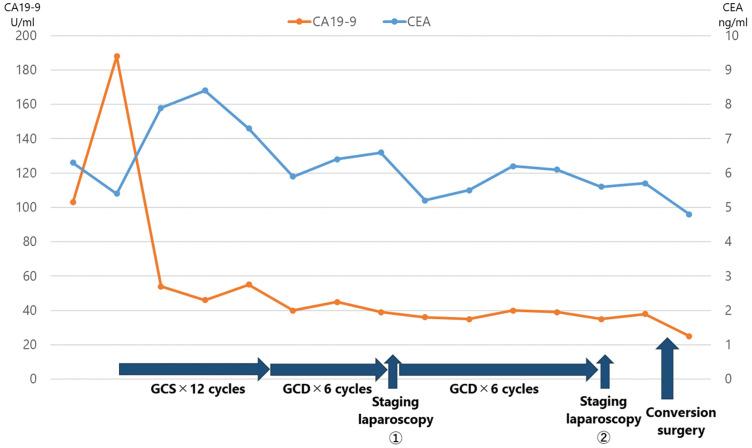
All the tumor marker levels decreased to within the normal ranges. CA19-9, carbohydrate antigen 19-9; CEA, carcinoembryonic antigen; GCD, durvalumab in combination with gemcitabine and cisplatin; GCS, S-1 in combination with gemcitabine and cisplatin

**Fig. 3:**
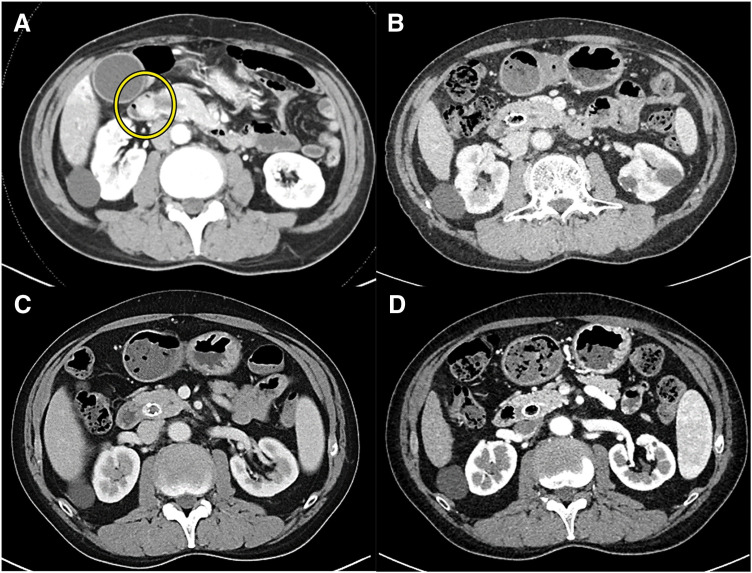
(**A**) CT image of the primary tumor at initial diagnosis. Yellow circle, tumor. (**B**) CT image of the primary tumor after 12 cycles of GCS. (**C**) CT image of the primary tumor before the first staging laparoscopy. (**D**) CT image of the primary tumor before conversion surgery. GCS, S-1 in combination with gemcitabine and cisplatin

**Fig. 4:**
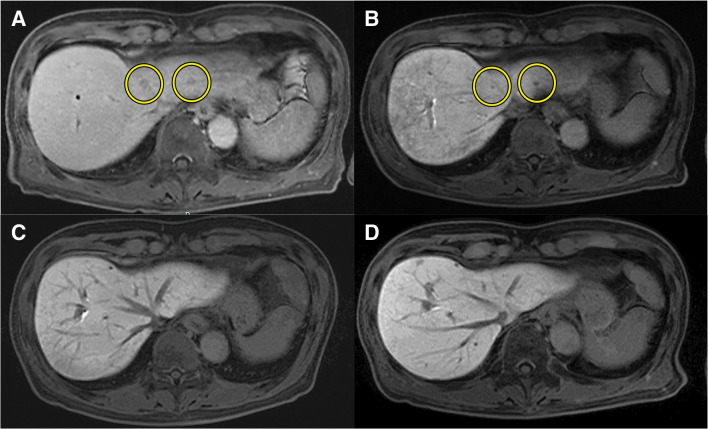
(**A**) EOB-MRI images obtained at initial diagnosis. Yellow circles, liver metastases. (**B**) EOB-MRI images obtained after 12 cycles of GCS. Yellow circles, liver metastases. (**C**) EOB-MRI images obtained prior to the first staging laparoscopy. (**D**) EOB-MRI images obtained before conversion surgery. EOB-MRI, gadolinium ethoxybenzyl diethylenetriamine pentaacetic acid-enhanced magnetic resonance imaging; GCS, S-1 in combination with gemcitabine and cisplatin

**Fig. 5:**
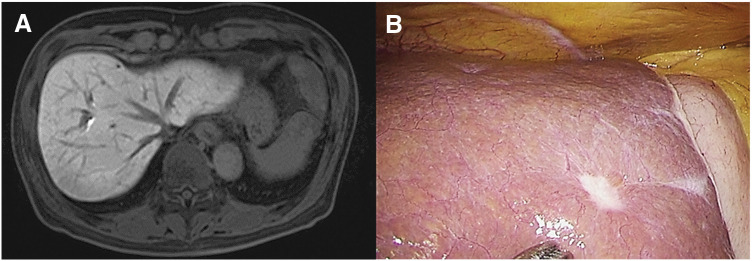
(**A**) EOB-MRI obscured liver metastases before staging laparoscopy. (**B**) Staging laparoscopy was performed, and adenocarcinoma was found on the liver surface. EOB-MRI, gadolinium ethoxybenzyl diethylenetriamine pentaacetic acid-enhanced magnetic resonance imaging

There were no obvious liver metastases or peritoneal dissemination intraoperatively. Abdominal cytology was negative. Although the patient had undergone chemotherapy, tissue adhesion was mild (**Fig. 6**). Pathological examination revealed that the primary lesion had a complete response (**Fig. 7A** and **7B**). There were no obvious metastases in the dissected lymph nodes (**Fig. 7C**), and R0 resection was achieved. In this case, tissue from the primary lesion, which was collected during a preoperative endoscopic US-guided procedure was stained with a primary antibody against PD-L1 (SP263 clone; Roche, Basel, Switzerland). The PD-L1 tumor area positivity score, which is the percentage of tumor cells stained with PD-L1 regardless of intensity, revealed a PD-L1 expression of less than 1% (**Fig. 8A** and **8B**).

**Fig. 6:**
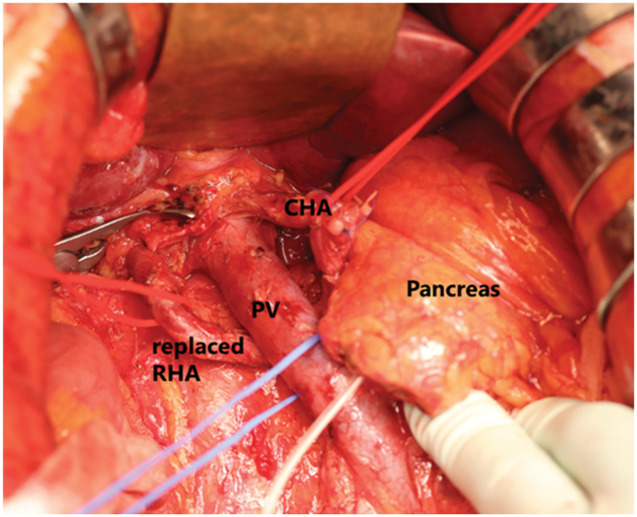
Intraoperative findings revealed mild tissue adhesion. CHA, common hepatic artery; PV, portal vein; RHA, right hepatic artery

**Fig. 7:**
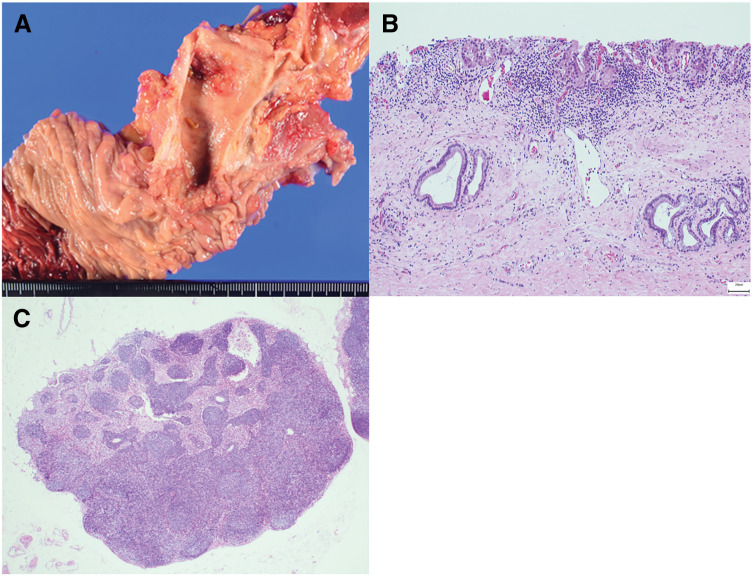
(**A**) Macroscopic findings of the resected specimen revealed that the gross malignancy was unclear. (**B**) There was no residual disease in the primary tumor in the resection specimen (hematoxylin and eosin staining, ×200). (**C**) There were no obvious metastases in the dissected lymph nodes (hematoxylin and eosin staining, ×200).

**Fig. 8:**
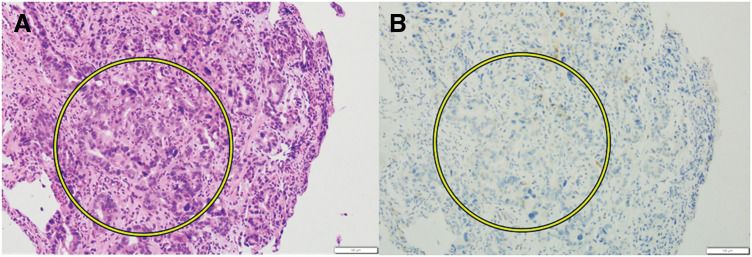
(**A**) Histopathological findings of preoperative biopsy tissue from the ampulla of Vater carcinoma, indicating adenocarcinoma (hematoxylin–eosin stain, ×40). Yellow circle, tumor area. (**B**) There was no obvious positive PD-L1 expression in the tumor cells (SP263 clone; Roche, ×40). Yellow circle, tumor area. PD-L1, programmed death ligand-1

The patient was scheduled to start postoperative adjuvant chemotherapy with GCD, which had been effective preoperatively, but the initiation was discontinued because of dehydration and poor nutrition associated with anorexia. Five months after surgery, there was an increase in tumor marker levels and recurrence in hepatic segment 8 (**Fig. 9A** and **9B**). The segment 8 liver lesion represented a newly developed postoperative metastasis rather than regrowth of a previously treated lesion.

**Fig. 9:**
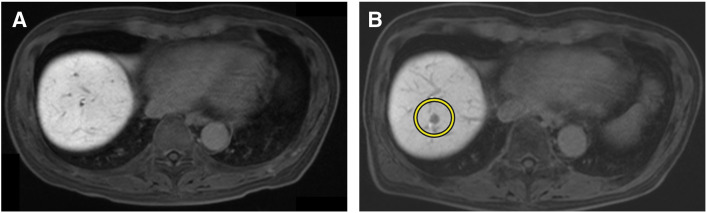
(**A**) EOB-MRI images obtained before conversion surgery in S8. No obvious liver metastases were observed. (**B**) EOB-MRI images taken 5 months after the operation revealed a new metastatic lesion in S8. Yellow circle, metastatic tumor. EOB-MRI, gadolinium ethoxybenzyl diethylenetriamine pentaacetic acid-enhanced magnetic resonance imaging; S8, segment 8 of the liver

Nutritional status was restored, and post recurrence chemotherapy was started 6 months after surgery. GCD therapy was restarted, and tumor marker levels decreased. Eighteen months after surgery, GCD was continued, with no evidence of local recurrence or new liver metastases. The treatment response was considered a stable disease.

## DISCUSSION

Notably, in this case, an ampulla of Vater carcinoma previously deemed unresectable became amenable to radical resection because of regression of distant metastatic lesions. Yabushita et al.^9)^ reported that, compared with chemotherapy alone, conversion surgery provides superior long-term outcomes in patients with locally advanced, unresectable biliary tract cancer. However, since this clinical study excluded patients with distant metastasis, the reports of achieving curative resection via conversion surgery for biliary tract cancer with distant metastasis, as in this case, are highly novel.

The ampulla of Vater carcinoma was not included in the international clinical trial TOPAZ-1,^6)^ and there is no direct evidence regarding the additive effect of durvalumab. Conversely, ampulla of Vater carcinoma is classified as a subtype of biliary tract cancer in Japan.^10)^ As with other types of biliary tract cancers, GC and GS therapies have been used as standard treatments for ampulla of Vater carcinoma.^10)^ Based on these considerations, we administered GCD therapy in this case in line with the evidence established for biliary tract cancer.

This case suggests the potential efficacy of GCD therapy for unresectable ampulla of Vater carcinoma. It may also serve as a valuable reference when evaluating future ampulla of Vater carcinoma treatment strategies. Given this background, we also considered preoperative evaluation, the role of GCD therapy, biomarkers, and postoperative chemotherapy.

We emphasized the importance of staging laparoscopy as a preoperative evaluation. At our institution, staging laparoscopy is primarily performed to assess residual lesions on the liver surface. However, if preoperative imaging makes it difficult to determine the presence of deep intrahepatic lesions, intraoperative ultrasonography is used for further evaluation. In the present case, preoperative imaging clearly demonstrated the absence of deep intrahepatic lesions; therefore, residual tumors on the liver surface were evaluated using staging laparoscopy alone. Although staging laparoscopy is rarely performed routinely in cases of biliary tract cancer, its usefulness has been reported.^11–15)^ It is particularly useful for confirming the histologic disappearance of metastases in patients with distant metastases of biliary tract cancers.^11–15)^ Hashimoto et al.^16)^ performed a retrospective study of 236 patients with biliary tract cancer who were scheduled for surgery and found unresectable factors after laparotomy in 11% of all cases. These cases were abandoned at exploratory laparotomy. Peritoneal dissemination was the most common unresectable factor in 9 patients (34.6%), followed by liver metastasis in 7 patients (26.9%).^16)^ In this case, although imaging studies confirmed the disappearance of liver metastases, staging laparoscopy was actually performed to detect microscopic metastases on the liver surface.

The importance of tumor markers has been suggested for the preoperative evaluation of biliary tract cancer. In 2023, Nakamura et al.^17)^ investigated prognostic factors in 246 patients with unresectable biliary tract cancer, including ampulla of Vater carcinoma, who received GC or GCS therapy. They compared outcomes between patients who underwent conversion surgery and those who did not.^17)^ The study demonstrated that elevated preoperative CA19-9 levels were a significant adverse prognostic factor, suggesting that normalization of CA19-9 prior to surgery is important when determining eligibility for conversion surgery.^17)^ In this case, the levels of all tumor markers decreased after the initial visit, and conversion surgery was performed.

There have already been 2 reported cases of conversion surgery for initially unresectable ampulla of Vater carcinoma (**Table 1**).^18,19)^ In both cases, the decision to proceed with conversion surgery was based on radiologically confirmed tumor shrinkage and persistently negative tumor marker levels.^18,19)^ Based on our case as well, we also believe that tumor shrinkage, as confirmed by preoperative imaging, and normalization of tumor markers are appropriate criteria for determining eligibility for conversion surgery. Furthermore, staging laparoscopy may also be useful as a criterion for determining the need for conversion surgery in cases in which lesions need to be assessed, such as those with liver metastases or peritoneal seeding.

**Table 1:** A case series of conversion surgery for unresectable ampulla of Vater carcinoma

Author	Year	Age	Sex	Unresectable factor	Duration of chemotherapy (months)	Time from diagnosis to conversion surgery (months)	Postoperative chemotherapy	DFS (months)
Fujii et al.^18)^	2016	53	M	Lymph node around the aorta (#16a2, #16b1) metastases	GC (2 months)	5.5	None	36
Sato et al.^19)^	2019	68	F	Lymph node #14d and liver metastases	GC (19 months)	21	None	10
Our case	2025	69	M	Liver metastases	GCS → GCD (16 months)	17	GCD	5

DFS, disease-free survival; F, female; GC, gemcitabine + cisplatin; GCD, durvalumab in combination with gemcitabine and cisplatin, GCS, S-1 in combination with gemcitabine and cisplatin; M, male

In the present case, GCD therapy was administered in combination with GCS therapy, and the role of GCD therapy warrants further evaluation. Tomino et al.^20)^ reported a case in which the sequential administration of GCS and GCD therapies for unresectable hilar cholangiocarcinoma resulted in a pathological complete response and conversion surgery. As in our case, downstaging was achieved through the sequential administration of GCS and GCD therapies. This suggests that the treatment strategy of first achieving tumor shrinkage with GCS therapy, followed by GCD therapy for long-term efficacy, contributed significantly to the success of conversion surgery.

However, the most appropriate first-line chemotherapy regimen remains open to debate, especially when conversion surgery is also being considered. GCS therapy demonstrated a higher response rate than GCD therapy (41% vs. 26.7%) and showed strong tumor shrinkage effects.^6,7)^ It seems sensible to first try to shrink the tumor with GCS therapy as a first-line chemotherapy. In contrast, the efficacy of GCD therapy as first-line chemotherapy needs to be evaluated. We are looking forward to the results of future clinical trials, which will help us to develop treatment strategies using GCD therapy.

In a study by Nakamura et al.,^17)^ conversion surgery was successfully performed in 8 cases of unresectable biliary tract cancer. The median number of cycles administered in the initial regimen was 10 (range, 1–12), and the median time to conversion surgery was 9 months (range, 6–19 months).^17)^ Based on our case and the previous report, we consider it appropriate to administer approximately 10–12 cycles of the initial regimen. We then administered GCD therapy, and after confirming tumor shrinkage and negative tumor markers, we performed conversion surgery. Although the optimal duration of preoperative treatment including GCD therapy for conversion surgery in patients with unresectable biliary tract cancer is unclear, we believe that a long-term treatment plan involving multiple regimens is necessary.

Although the TOPAZ-1 trial is investigating PD-L1 as a biomarker of durvalumab efficacy, validation of biomarkers, including PD-L1, has not yet been conducted in ampulla of Vater carcinoma. In this case, less than 1% of the tumor area was positive for PD-L1. The cutoff value for PD-L1 expression in the TOPAZ-1 trial was 1%.^6,21)^ Thus, the patient was diagnosed with PD-L1-negative ampulla of Vater carcinoma. The efficacy of GCD in patients undergoing conversion surgery for unresectable gallbladder cancer has been reported, regardless of PD-L1 positivity or negativity.^22,23)^ The use of anti-PD-L1 antibody drugs in conversion surgery for unresectable biliary tract cancer has rarely been reported, and the efficacy of evaluating PD-L1 expression remains unclear. In particular, the TOPAZ-1 trial revealed no correlation between PD-L1 expression and therapeutic response in biliary tract cancers.^6)^ In the future, the establishment of biomarkers to predict the efficacy of PD-L1 antibody drugs will be needed.

Given the high postoperative recurrence rate of biliary tract cancer, postoperative therapy for resectable disease has become increasingly standardized.^24)^ However, adjuvant therapy following conversion resection after preoperative treatment for unresectable biliary tract cancer has yet to be established. In the present case, GCD therapy was adopted because it resulted in a pathological complete response after tumor shrinkage plateaued with GCS therapy. There is a clinical rationale for using drugs from the same class to target microscopic residual tumors postoperatively. We also considered the tail-plateau effect of immune checkpoint inhibitors and expected that durvalumab would offer long-term disease control.^6,25)^ In addition, good disease control was observed even after the disease recurred. The patient survived for 18 months after surgery, which is considered a long survival period for ampulla of Vater carcinoma with distant metastasis. These findings suggest that continued GCD therapy may be beneficial.

## CONCLUSIONS

This report described the successful treatment of an unresectable ampulla of Vater carcinoma with sequential systemic chemotherapy including durvalumab. Radical resection was achieved through conversion surgery. In contrast, regarding treatment strategies aimed at conversion surgery for unresectable biliary tract cancer, including the ampulla of Vater carcinoma, it remains unclear whether GCD is more effective as a first-line therapy or as a sequential therapy. Based on this case, we believe that sequential administration of GCS followed by GCD may contribute to achieving conversion surgery for unresectable ampulla of Vater carcinoma. GCD therapy is expected to be a useful treatment option in the future to help achieve conversion surgery.

## DECLARATIONS

### Availability of data and materials

The datasets generated and/or analyzed during the current case report are not publicly available due to patient confidentiality, but are available from the corresponding author on reasonable request.
